# The study of routine laboratory factors in children with mycoplasma pneumoniae pneumonia: serum uric acid may have anti‐inflammatory effect

**DOI:** 10.1002/jcla.24026

**Published:** 2021-10-16

**Authors:** Chenglin Pan, Yanjie Chen, Shaosheng Wang, Ming Li, Shen Qu

**Affiliations:** ^1^ Department of Pediatrics School of Medicine Shanghai Tenth People’s Hospital Tongji University Shanghai China; ^2^ Department of Laboratory Medicine Maternity Service Center of Pengzhou Maternal & Child Health Care Hospital Chengdu China; ^3^ Department of Pediatrics Hainan Maternal and Children’s Medical Center Haikou Hainan China; ^4^ Department of Endocrinology & Metabolism School of Medicine Shanghai Tenth People's Hospital Tongji University Shanghai China

**Keywords:** anti‐inflammatory, children, laboratory factors, mycoplasma pneumoniae pneumonia, serum uric acid

## Abstract

**Background:**

High uric acid levels are a risk factor for cardiovascular disorders, and metabolic diseases; however, the role of serum uric acid (sUA) during the mycoplasma pneumoniae pneumonia (MPP) of children is poorly known. This study aimed to clarify the effects of sUA during the MPP of children.

**Methods:**

This was a prospective cohort study of children with MPP from multi‐center inpatient departments from September 2019 to August 2020. Routine laboratory characteristics analyzed including ALT, AST, BUN, CREA, UA, LDH, CK‐MB, WBC, N%, PLT, and CRP. Subjects were divided into 3 groups: non‐MPP, mild MPP (MMPP), and severe MPP (SMPP).

**Results:**

949 subjects were enrolled, including 207 in non‐MPP, 565 in MMPP, and 177 in SMPP. The optimal cutoff value for sUA is 239 μmmol/L in receiver operating characteristic (ROC) curves analysis. Multivariate logistic regression showed that WBC and sUA had significance for protective effects between non‐MPP and SMPP, but CRP did not have significance between the two groups, N and PLT had significance for risk factors; WBC and sUA did not have significance for the protective effects between non‐MPP and MMPP, CRP had significance between the two groups, N and PLT had significance for the risk effects. Similarly, binary logistic regression showed UA, WBC, and CRP had significance for the protective effects between non‐MPP and MPP, but N and PLT had significance for the risk effects between the two groups.

**Conclusion:**

Both multivariate and binary logistic regression demonstrated that sUA displayed a protective effect during the MPP of children, which meant sUA is anti‐inflammatory.

## Graphical abstract

High uric acid levels are a risk factor for cardiovascular disorders,metabolic diseases; however, the present study displayed that serum uric acid (sUA) during the mycoplasma pneumoniae pneumonia (MPP) of children may have anti‐inflammatory effect.

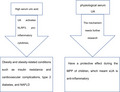

## 1 INTRODUCTION

Uric acid (UA), the product of purine catabolism, is a damage‐associated molecular pattern (DAMP) released from ischemic tissues and dying cells.^1^, ^2^ Primates include humans lost the uricase gene, and the direct consequence is that they have higher serum uric acid levels than other animals.^3^ The mutation in the uricase gene that occurred during food scarcity and global cooling resulted in a survival advantage at that time.^4^ Today, however, it is associated with hypertension, kidney disease, obesity, and diabetes.^5^ Previous studies reported that UA can activate the NLRP3 (NLR family, pyrin domain containing 3) inflammasome.^6^, ^7^ Upon NLRP3 inflammasome activation, cells secrete increased amounts of pro‐inflammatory cytokines, such as IL‐1β and IL‐18,^8^ which subsequently accelerates the development of obesity,^9^ and obesity‐related conditions such as insulin resistance and cardiovascular complications,^9^, ^10^, ^11^ type 2 diabetes,^12^ and NAFLD.^13^, ^14^, ^15^ NLRP3 is not only associated with sterile inflammation, but also associated with pathogenic infections. Mycoplasma pneumoniae is an atypical bacterial respiratory pathogen known to cause a range of airway inflammation and lung and extrapulmonary pathologies. Segoviady et al. reported that M. pneumoniae infection activates the NLRP3 inflammasome complex, leading to IL‐1 secretion, inflammation, and innate immune cell activation in the lungs of infected C57BL/6 mice and in mouse bone marrow‐derived macrophages (BMDMs).^16^

In the process of evolution, humans have lost the capacity to synthesize uricase.^17^ For this reason, humans possess tenfold higher concentrations of serum UA than mice (180–400 μΜ vs. 18–40 μΜ).^18^, ^19^ TarcioTeodoro Braga et al. also found that 180 μΜ soluble UA, rather than displaying inflammatory properties, may present an anti‐inflammatory activity in human macrophages.^20^ Jenn‐Haung Lai et al. also reported that physiological concentrations of soluble UA displayed anti‐inflammatory and chondroprotective effects.^21^ Therefore, we sought to investigate that sUA had a protective or risk effect during the *M. pneumoniae* infection of children. The result of the study showed that sUA during MPP of children had a protective effect on the body, which mechanism is needed to be further studied.

## 2 MATERIALS AND METHODS

### 2.1 Subjects

Research data were collected from multi‐center inpatient departments, including Shanghai Tenth People's Hospital, School of Medicine, Tongji University; Hainan Maternal and Children's Medical Center; Maternity Service Center of Pengzhou Maternal and Child Health Care Hospital; Huai'an First People's Hospital, Nanjing Medical University. We performed a retrospective analysis of medical records of patients with non‐MP pneumonia and MPP (mild MPP and severe MPP). Non‐MPP included upper respiratory infection and bronchitis of children. The classification of mild and severe pneumonia was referred to this.^22^ The diagnosis of MP infection was based on the positive results for a serologic test (MP IgM positive and antibody titer 1:160) while having the positive results for MP polymerase chain reaction (PCR) tests of nasopharyngeal secretions. This study was approved by the Ethics Committee of Shanghai Tenth People's Hospital. All of the procedures performed in studies involving human participants were in accordance with the ethical standards of the national guidelines. The approval number is NCT04568232.

### 2.2 Data collection

Laboratory data were retrospectively collected from all children who were included in the study. Characteristics analyzed liver function markers, including alanine aminotransferase (ALT), aspartate aminotransferase (AST) and renal function [blood urea nitrogen (BUN), creatinine (CREA), and uric acid(UA)], lactate dehydrogenase (LDH), creatine kinase MB (CK‐MB), white blood cell (WBC), neutrophil(%), platelet(PLT), and C‐reactive protein (CRP). Venous blood samples were collected from the subjects after they were fasted for at least 10h in the morning. The study was approved by the Ethics Committee of Shanghai Tenth People's Hospital, School of Medicine, Tongji University, and the data from patients were analyzed anonymously.

### 2.3 Statistical Analysis

All data were analyzed using SPSS 20 (IBM), and *p *< 0.05 was considered statistically significant. Receiver operating characteristic (ROC) curves were used to analyze the optimal cutoff value of uric acid. Normally distributed data were reported as mean ± standard deviation. One‐way ANOVA was used to compare these data. Data with a skewed distribution were presented as median values (interquartile). The comparisons were made by Kruskal‐Wallis test. Binary logistic regression was used to analyze protect or risk effects of laboratory factors between non‐MPP and MPP. Protect or risk effects of laboratory characteristics were made by multiple logistic regression among non‐MPP, MMPP, and SMPP.

## 3 RESULTS

Table 1 showed that the main clinical features and laboratory values. The age was older in the SMMP samples than that in the non‐MPP and MMPP, and the result was consistent with the previous report.^23^ There was no significant difference in sex distribution among the 3 groups (*p *= 0.317). We also found that thermal spike, Fever >7 days, range of lung invasion, Pleural effusion, extrapulmonary complications, PLT, CRP, ALT, AST, LDH, BUN, CREA, and UA had significance; but WBC and CK‐MB had no significance among the 3 groups (Table 1). The optimal cutoff value for uric acid is 239 μmmol/L in ROC analysis (AUC: 0.575, 95% CI: 0.511–0.638). The results of binary regression analysis of the laboratory characteristics are shown in Table 2. On the basis of binary regression analysis, we found that ALT (*p *= 0.949, OR = 0.999, 95% CI (0.959–1.040)), AST (*p *= 0.468, OR = 0.985, 95% CI (0.947–1.025)), BUN (*p *= 0.233, OR = 0.832, 95% CI (0.614–1.126)), CREA(*p *= 0.998, OR = 1.000, 95% CI (0.953–1.049)), CK‐MB (*p *= 0.285, OR = 0.986, 95% CI (0.961–1.012)), and LDH (*p *= 0.165, OR = 1.003, 95% CI (0.999–1.007)) did not have significance between non‐MPP and MPP; but WBC (*p *= 0.010, OR = 0.895, 95% CI (0.823–0.973)), UA (*p *= 0.024, OR = 0.417, 95% CI (0.195–0.891)), and CRP (*p *= 0.026 OR = 0.980, 95% CI (0.963–0.998)) were significant for protective factors between the two groups; N (*p *= 0.004, OR = 1.038, 95% CI (1.012–1.065)) and PLT (*p *= 0.002, OR = 1.006, 95% CI (1.002–1.011)) were significant risk factors. The results of multiple regression analysis of the associated factors in non‐MPP, mild MPP, and severe MPP are shown in Table 3. The Table 3 implied that WBC (*p *= 0.050, OR = 0.908, 95% CI (0.824–1.000)) and UA (*p *= 0.067, OR = 0.469, 95% CI (0.209–1.054)) did not have significance for protective effect between non‐MPP and MMPP, but CRP (*p *= 0.007, OR = 0.970, 95% CI (0.948–0.991)) was a significant protective factor between the two groups, N (*p *= 0.035, OR = 1.030, 95% CI (1.002–1.058)) and PLT (*p *= 0.007, OR = 1.007, 95% CI (1.002–1.011)) were significant risk factors between the two groups; Table 3 also uncovered that WBC (*p *= 0.003, OR = 0.839, 95% CI (0.749–0.941)) and UA (*p *= 0.009, OR = 0.286, 95% CI (0.111–0.736)) had significance for the protective effect between non‐MPP and SMPP, which signified that sUA had significant protective factor between MMPP and SMPP, N (*p *≤ 0.001, OR = 1.062, 95% CI (1.030–1.096)) and PLT (*p *= 0.002, OR = 1.007, 95% CI (1.003–1.012)) were significant for risk factors, but CRP (*p *= 0.213, OR = 0.987, 95% CI (0.968–1.007)) did not have significance between non‐MPP and SMPP. Multiple regression analysis also showed that ALT (*p *= 0.465, OR = 0.983, 95% CI (0.941–1.028)), AST (*p *= 0.929, OR = 0.998, 95% CI (0.957–1.041)), BUN (*p *= 0.486, OR = 0.885, 95% CI (0.628–1.248)), CREA (*p* = 0.293, OR = 1.029, 95% CI (0.976–1.084)), LDH (*p* = 0.971, OR = 1.000, 95% CI (0.995–1.005)), and CK‐MB (*p* = 0.717, OR = 0.995, 95% CI (0.968–1.023)) did not have significance between non‐MPP and SMPP; ALT (*p* = 0.369, OR = 1.022, 95% CI (0.974–1.073)), AST (*p* = 0.231, OR = 0.971, 95% CI.

**TABLE 1:** Comparison of patients with non‐MPP, MMPP, and SMPP

Parameter	Control	MMPP	SMPP	*p*
Clinical information				
Age (year)	2 (3)	4 (4)	5 (4)	≤0.001
Sex (male/female)	132/99	277/264	93/83	0.317
Thermal spike^a^	3 (1)	0 (3)	3 (1)	0.002
Fever>7 days^b^	0 (0)	0 (0)	1 (1)	≤0.001
Range of lung invasion^c^	0 (0)	1 (0)	2 (0)	≤0.001
Pleural effusion (1/3)^d^	0 (0)	0 (0)	1 (1)	≤0.001
Extrapulmonary complications^e^	0 (0)	0 (0)	1 (1)	≤0.001
Laboratory values				
WBC (*10^9^/L)	8 (4.33)	7.1 (3.3)	7.4 (3.8)	0.085
N (%)	41 (34.25)	53 (22)	57 (21)	≤0.001
PLT (*10^9^/L)	278.5 (146.5)	322 (157.5)	335 (169)	≤0.001
CRP (mg/L)	4.36 (13.46)	12.3 (20.25)	15.935 (28.53)	≤0.001
ALT (IU/L)	14 (13)	14 (7.5)	15.5 (12.0)	≤0.001
AST (IU/L)	36 (20)	32 (13)	34 (18)	≤0.001
LDH (IU/L)	291 (89)	287 (93.5)	325 (131.5)	0.020
CK‐MB (IU/L)	26 (15)	25 (13)	22.5 (11)	0.082
BUN (mmol/L)	3.74 (1.58)	3.28 (1.2)	2.775 (1.63)	≤0.001
CREA (μmol/L)	29.6 (6.9)	29.1 (10.25)	28.5 (10.5)	≤0.001
UA (μmol/L)	264.469 ± 92.793	236.1763 ± 65.51937	221.7881 ± 75.28576	≤0.001

Abbreviations: ALT, alanine aminotransferase; AST, aspartate transaminase; BUN, blood urea nitrogen; CK‐MB, creatine kinase isoenzyme; CREA, creatinine; CRP, C‐reactive protein; LDH, lactic dehydrogenase; N, neutrophil ratio; PLT, platelet; UA, uric acid; WBC, white blood cell.

^a^ (0: normal, 1: low heat, 2: moderate heat, 3: hyperpyrexia, 4: ultra‐hyperpyrexia)

^b^ (0: <7 days; 1: >7 days).

^c^ (<=1/3 is mild; 1: multiple lobar involvement are severe).

^d^ (0: no; 1: yes).

^e^ (0: no; 1: yes).

**TABLE 2:** Binary logistic regression analysis of associated characteristics in non‐MPP and MPP (include mild and severe MPP)

Laboratory characteristics	OR	*p*	95% CI
White blood cell (WBC)	0.895	0.010	0.823–0.973
Neutrophil (%)	1.038	0.004	1.012–1.065
Platelet (PLT)	1.006	0.002	1.002–1.011
C‐reactive protein (CRP)	0.980	0.026	0.963–0.998
Uric acid (UA)	0.417	0.024	0.195–0.891

**TABLE 3:** Multiple logistic regression analysis of associated factors in non‐MPP, MMPP, and SMPP)

Laboratory characteristics	MMPP			SMPP		
	OR	P	95% (CI)	OR	P	95%(CI)
WBC	0.908	0.050	0.824–1.000	0.839	0.003	0.749–0.941
Neutrophil (%)	1.030	0.035	1.002–1.058	1.062	≤0.001	1.030–1.096
Platelet (PLT)	1.007	0.003	1.002–1.011	1.007	0.002	1.003–1.012
CRP	0.970	0.007	0.948–0.991	0.987	0.213	0.968–1.007
Uric acid (UA)	0.469	0.067	0.209–1.054	0.286	0.009	0.111–0.736

(0.925–1.019)), CREA (*p* = 0.292, OR = 0.968, 95% CI (0.911–1.028)), CK‐MB (*p* = 0.330, OR = 0.984, 95% CI (0.954–1.016)) did not have significance between non‐MPP and SMPP; but BUN (*p* = 0.026, OR = 0.636, 95% CI (0.427–0.947)) had a significance for the protective effect between non‐MPP and SMPP; LDH (*p* = 0.014, OR = 1.006, 95% CI (1.001–1.010)) was a risk factor between non‐MPP and SMPP.

## 4 DISCUSSION

Uric acid (UA) is the end product of purine metabolism in humans due to the loss of uricase activity by various mutations of its gene during the Miocene epoch, which led to humans having higher UA levels than other mammals. Furthermore, 90% of UA filtered by the kidneys is reabsorbed, instead of being excreted. These facts suggest that evolution and physiology have not treated UA as a harmful waste product, but as something beneficial that has to be kept. This has led various researchers to think about the possible evolutionary advantages of the loss of uricase and the subsequent increase in UA levels. It has been argued that due to the powerful antioxidant activity of UA, the evolutionary benefit could be the increased life expectancy of hominids, maintain blood pressure in times of very low salt ingestion, and have higher intelligence in humans.^24^ Some researchers reported that hyperuricemia is the primary risk factor for developing gout, hypertension, renal disease, metabolic syndrome, diabetes, and cardiovascular disease, but how is the function of sUA during the MPP of children? NLRP3 is a critical regulator of inflammation, which M. pneumonia infection activates the NLRP3 inflammasome complex, leading to IL‐1 secretion, the MyD88/NF‐κB signaling pathway was essential for increasing gene expression of pro‐IL‐1 and IL‐6 during mycoplasma pneumonia infection. Soluble uric acid exerts an inflammation‐stimulatory effect and induced the production of tumor necrosis factor α (TNFα), interleukin (IL)‐6, and IL‐1β,^25^ it also involved in the lung injury, COPD (chronic obstructive pulmonary disease), and EP (eosinophilic pneumonia).^26^, ^27^, ^28^

But, according to binary regression analysis in the present study, we found that sUA, WBC, and CRP have significance for the protective effect between non‐MPP and MPP groups, which meant they involved in the anti‐inflammatory activity, but the OR value of CRP is proximal 1, so available protection is small. On the base of multiple logistic regression, sUA, WBC, and CRP did not have significance for protection between non‐MPP and MMPP, which suggested that sUA and WBC had protection between the two groups, but the value of OR of WBC and CRP also was close to 1, so their protection is small; sUA and WBC had significance for the protection between non‐MPP and SMPP, which it showed that sUA and WBC have anti‐inflammation between non‐MPP and SMPP, and CRP was not significant between the two groups.

N and PLT were risk factors for MPP. Platelets have long been recognized as the major blood cells for activation of the coagulation system, and it can influence the different cell types including T‐lymphocytes, neutrophils, mononuclear phagocytes, endothelial cells, and dendritic cells, activated platelets and could trigger inflammation.^29^, ^30^ In addition, neutrophils promote intravascular blood coagulation and thrombosis during infections and inflammatory responses.^31^ One of the risks of Neutrophil and platelets during MPP of children is associated with thrombosis. In the present study, there was a significance among non‐MPP, MMPP, and SMPP, which was in line with the previous study.^32^

Qiuyue Ma et al. also identified that soluble uric acid has anti‐inflammatory effects on activated monocytes.^33^ The present study had also shown that sUA had a protective effect during MPP of children. This may be one of the reasons that humans lost enzyme uricase during human evolution. When humans suffered from stress and disease such as MPP, the human can respond by mobilizing multiple systems and organs of the whole body to deal with it, increasing the concentration of serum uric acid, especially SSMP to protect the body. MPP especially SMPP is prone to have extrapulmonary complications through direct invasion and immune impairment, which results in transient increasing concentration of sUA, and increases the anti‐inflammation effect. If the sustained elevation of sUA will have harmful effects on the body such as hypertension, kidney disease, obesity, diabetes, and gout, after the recovery from SMPP, the concentration of uric acid gradually returns to normal, which whether it maintains a minimal level of anti‐inflammation needs to be further researched. Our findings also raised the possibility that sUA level may serve as a useful biomarker for SMPP, which is consistent with the study result in severe malaria.^34^

The secretion of CRP starts within 4–6 h, and its level doubles every 8 h; it then reaches its maximum level within 36–50 h. After the stimulation is removed, the CRP level falls relatively quickly, with a half‐life of 19 h.^35^ WBC counts and neutrophil ratios and CRP are easily affected by other factors, such as coinfection with bacteria, the use of glucocorticoids, and the duration of infection. Therefore, their effects must be analyzed together with other indicators. The present study had several limitations. First, it was a retrospective observational study, and therefore, there may have been some selection bias. Secondly, there are some cases in which the patients had a combined MP and other pathogens infection which cannot be detected. Thirdly, the number of SMPP is not very enough.

In conclusion, we found that sUA has a protective effect during the MPP of children, which suggested it had the anti‐inflammatory activity in the present study. The results suggested that urease loss may be one of the causes during human evolution. Further research needs to do determine whether NLRP3 and other signaling pathways are responsible for this anti‐inflammation and whether physiological sUA is part of innate immune molecules.

## CONFLICTS OF INTEREST

The authors declare that they have no conflicts of interest.

## Data Availability

The data that support the study are available from the corresponding author upon reasonable request.
